# An Evaluation of Pumping Stations for Pressure Sewers System Made from Concrete Coils, Polymer Concrete, and High-Density Polyethylene (HDPE)

**DOI:** 10.3390/ma16020524

**Published:** 2023-01-05

**Authors:** Tomasz Sionkowski, Wiktor Halecki, Krzysztof Chmielowski

**Affiliations:** 1 Grundfos Pompy Sp. z.o.o., Klonowa 23, 62-081 Baranowo, Poland; 2 Institute of Nature Conservation, Polish Academy of Sciences, Mickiewicza 33, 31-120 Krakow, Poland; 3 Department of Sanitary Engineering and Water Management, Faculty of Environmental Engineering and Land Surveying, University of Agriculture in Krakow, Mickiewicza 21, 31-120 Krakow, Poland

**Keywords:** polymer material, sewage-treatment system, simulation model, waste management

## Abstract

A deficiency in accurate and current regulations, along with a lack of experience in sanitary construction, makes the installation of sewers challenging. Using models, it was determined that if the pumps were operated simultaneously, the service would last for a long time over the entire sewer system. With a daily sewage inflow of 468 dm^3^, the system was found to run 14.4–14.7% longer than expected at 100 pumping stations. Each month, the pressure-sensitive sewer system receives more than 51 min of extended service from the city’s central sewer system. Increasing wastewater inflow and the number of pumps decrease centrifugal pump capacity. In the study, the main findings were related to the number of pumps. With 100 centrifugal pumps simultaneously, the pressure-based system was most effective. An increase in operation time of 18.4–19.1% was observed over a period of 30 days and an average sewage inflow of 705 dm^3^ for each. In place of gravity sewerage, sewerage can be used. Pressure sewer systems should be designed in a way that addresses technical as well as economic concerns. Accordingly, this study indicates that pressure sewerage is a viable alternative to gravity sewerage in villages with scattered drinking water supplies.

## 1. Introduction

Sanitary engineering has been developing rapidly around the world in recent years. From catchment analysis of water storage tanks [1,2,3], to linear wastewater conveyance systems [4,5,6], to wastewater treatment processes and methods, detailed studies are being conducted on the supply, collection and treatment of water, wastewater and sewage [7,8,9,10]. Technology is not the only factor that determines the efficiency of a system, but also the material it is made from [11,12,13,14,15]. Pressure sewer systems have become widespread in the last few years in European Union countries. Pressure sewerage systems consist of suction valves installed in a manhole, where domestic wastewater flows [16]. Wastewater from one or more households is pumped through side sewers and the main sewer to a sewage treatment plant or expansion chamber in a pressure sewerage system [17]. Pressure sewerage systems use free-flow centrifugal pumps, centrifugal pumps with a cutting knife, or positive displacement pumps [18]. The use of a particular solution depends on field conditions and the amount of inflowing sewage. The analysis of how the pressure sewer system operates has been the subject of research by many authors worldwide [19,20]. Studies of small pressure systems have been used to draw general conclusions about all pressure systems [21]. Pumping stations are practically imperceptible to an increase in operating time caused by a reduction in capacity under the impact of another pumping station. Due to the complexity of the processes involved in pressure sewers, further research is required. The process is currently under construction. Gravity sewers in rural areas are questioned for many reasons, including the use of numerical methods and verification of previous studies [22,23,24]. A high groundwater level, a large capital expenditure, and restricted flexibility during the design and implementation process are the primary reasons. Limitations in technical, economic, and operational aspects pose the greatest challenges. The first systems were implemented decades ago, yet there are still some inaccuracies in numerous publications [25,26,27,28,29].

Engineering practice uses vent and aeration valves to improve pipeline suction. In addition to providing a better understanding of how centrifugal pumps work, these findings provide a broader view of pressure channels built onto them. Designing can be greatly aided by this knowledge. Common guidelines do not specify how many pumps can be connected to a manifold at the same time, which is an important consideration. In studies of equalization systems, pumps are often overlooked. Poor quality materials often result in problems during pressure sewerage construction. A novel aspect of the study is the use of centrifugal pumps in domestic wastewater treatment systems. The study also presents a new method for evaluating simultaneous performance pressure systems. Additionally, sewer users lack knowledge of their impact on the environment. As a result, we have established the following objectives:

(a) to demonstrate the performance of centrifugal pumps operating simultaneously at rural, domestic sewage pumping stations;
(b) to simulate pressurized sewage pumps;
(c) to calculate a pressurized sewage system’s operating time;
(d) to provide solutions to help prevent system leveling.

## 2. Materials and Methods

### 2.1. Study Object and Data for System Calibration

Our study area was located in the Modlniczka municipality near Krakow. The village is characterized by a high level of groundwater and flat terrain (central part), while hilly areas can be observed on the eastern side. The elevation amplitude between the highest point of the sewer route and the expansion well with is 30.9 m. The expansion well was located at an altitude of 239.00 m above sea level. A dense population, developed infrastructure, high groundwater levels, and lack of land for intermediate pumping stations in the center of the village have determined the use of a pressurized sewer system in the majority of these areas. The expansion well was reached by two subsystems consisting of 7476 m of pressure sewer network (subsystem S1) and 2776 m of network (subsystem S2), respectively. These subsystems were independent of each other. The subject of the study was the S1 subsystem located in the central part of the village. Pressure connections from individual pumping stations to the collecting sewer and the initial sections of the system were made from polyethylene pipes with an outside diameter of DN55 (with a total length of 1378 m). As more pumping stations were connected to the main pipeline, the amount of sewage to be pumped also increased. Following the spring thaw, it reaches several centimeters below ground level in places. It became necessary to apply a gradation of pipeline diameters from DN63 diameter (with a total length of 2367 m, through DN75 diameter with a total pipeline length of 1450 m) to DN90 diameter with a length of 1102 m. The final section of the DN110 diameter pipeline leading to the expansion manhole mainly through sparsely populated areas had a length of 1078 m.

**Figure 1:**
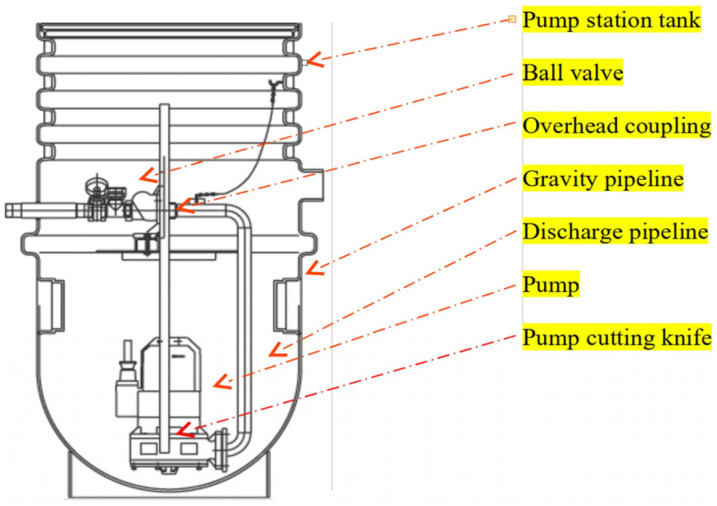
A functioning model of a pumping station.

The pressure sewer system in Modlniczka used domestic pumping stations made of high-density polyethylene with a total capacity of 890 dm^3^ and an internal diameter of 856 mm. The pumping stations were equipped with submersible pumps with a cutting knife from the Wilo TP40s/MTS series (Figure 1). The units were sized for flow rates ranging from 1.8 dm^3^/s to 3.5 dm^3^/s to ensure that the system achieves a periodic rate of self-cleaning of the pipelines. For the most part, 400-volt pumps were used. Only occasionally were single-phase 230-volt pumps with higher current ratings used. The applicable principle was one pumping well per property. Based on the field work in the hydraulic engineering laboratory, the sewer model was calibrated and the model was investigated.

### 2.2. Modeling of the Operation of the Pressure Sewerage System

**Figure A1:**
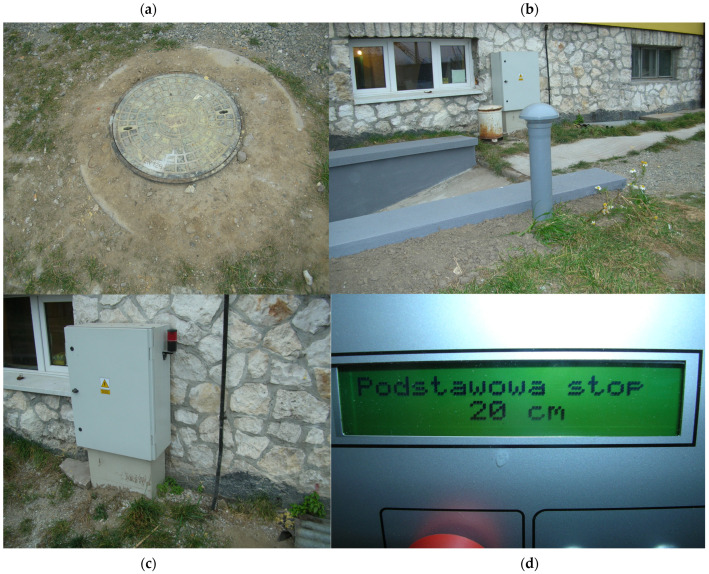
Pictures of field work while conducting measurements in the backyard of a residential house. (**a**) Pass-through manhole of the sewage pumping station; (**b**) View of the selected pumping station including the stack and control cabinet; (**c**) Control cabinet including optical indicator; (**d**) Controller—readout of pump shutdown level.

A hydraulic engineering laboratory pressurized sewer operation model was developed in the field (Figure A1). The sewage inflow and the degree of filling of the domestic pumping station with sewage were considered to be time-varying. The conceptual scheme of the sewerage system model is shown in Figure 1.

**Figure A2:**
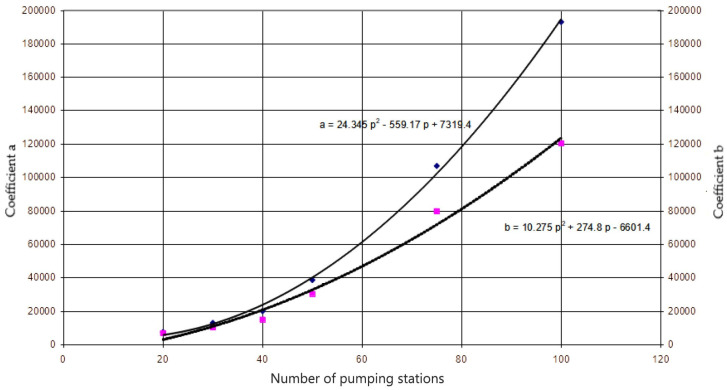
Relationships for functioning of working pumps.

Waterflow to the pumping station and pumping station operations are described by the following function (Figure A2):

$$f\left(Q_{\mathrm{pomp}}\left(t\right),Q\left(t\right),V\left(t\right){,d,h}_{\mathrm{emer}}{,h}_{\max}{,h}_{\min},h\left(t\right),v,V,t\right)=0$$

where:

Q_pomp(t)_—volume of wastewater pumped by the pump installed in the pumping station [dm/s].
V_(t)_—volume of wastewater in the pumping station at time t [dm^3^],
h_(t)_—change of wastewater in the pumping station at time t,
d—internal diameter of the pumping station [m],
v—velocity in the pressure pipe at the end of the system [m/s],
V—active volume of wastewater contained between h_max_–h_min_,
Q_(t)_—sewage inflow to the domestic pumping station at time t,
h_max_—maximum level of wastewater (level of activation of the pump) [m],
h_min_—minimum level of wastewater (pump off level) [m],
h_emer_—emergency height above the pump switch-on level [m].

**Figure A3:**
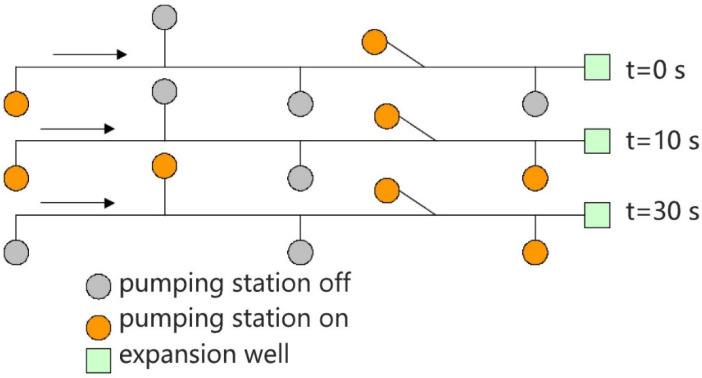
Schematic of pump function during simultaneous pressure system investigation.

A series of simulations (Figure A3) were conducted for systems consisting of 10, 20, 30, 40, 50, 75 and 100 domestic pumping stations. All simulations were carried out for two different levels of pump switching on and off. The pumps parameters and pumping stations in the two types of simulations were identical and are summarized in Table 1. In the simulations of Group 1 (Simulation 1), the pumping stations switched on more frequently, as the active volume of the pumping stations was almost twice lower (148.05 dm^3^) than the active volume for the Simulation 2 group (296.11 dm^3^).

**Figure A4:**
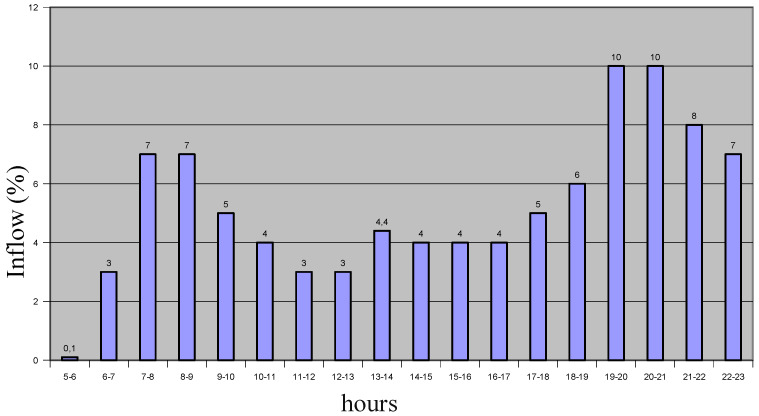
The dynamics of wastewater inflow to the pumping station by diurnal variation.

The dynamics of wastewater inflow to the pumping station was characterized by diurnal variation (Figure A4). The morning peak and the evening peak were most frequently observed. The simulation study assumed a daily distribution of wastewater inflow is characterized by two peaks: a smaller, morning peak from 7–10 a.m. and a larger, evening peak from 7–11 a.m. In the morning peak, 19% of the total daily wastewater discharge flowed into the system, and in the evening peak the value was 28%. The model acknowledged that the total percentage discharge of wastewater between 11 p.m. and 5 a.m. was 0.1%.

A time step of dt = del(t) = 10 s was used in all simulations. Simulations were carried out with daily random inflow of wastewater to the pumping station ranging from 250 dm^3^ to 600 dm^3^. Since the amount of inflow is a random variable, the volume of wastewater flowing into each manhole was randomized from the range given above, and the volume of wastewater flowing into each manhole was randomized. After running a series of simulations for a system consisting of k-number of pumping stations (where k was 10, 20, 30, 40, 50, 75, and 100 pumping stations, respectively), the daily volume of inflowing sewage was doubled on one randomly selected pumping station, followed by a series of simulations for a system consisting of k-number of pumping stations (each was connected to a single pump station). The procedure described above for increasing the amount of inflowing wastewater was applied to pumping stations included in a system with a k-number of pumping stations (all possibilities: 10, 20, 30, 40, 50, 75, and 100 pumping stations). The following simulation designations were introduced (Table 2):

SIM—simulation of pressure sewer operation (designation of pump switching levels, according to the parameters given in Table 1; the number of pumps involved in the simulation × 10 = 40 pumping stations per hour).

DOUBLE—the number of pumping stations for which the sewage inflow was doubled represents 50% of the pumping stations involved in the simulation, (for a system consisting of 75 pumping stations, the sewage inflow was increased to 38 of them).

Capacity costs for a gravity system where private houses are located 150 m (one from another, on average), and high levels of groundwater are 4 times higher than a pressured sewage system. Capacity costs for a gravity system where private houses are located 150 m (one from another, on average), and low levels of groundwater are 3 times higher than in a pressured sewage system. Operational costs of a pressurized sewage system consist of energy costs and maintenance costs (damages to pipes, damages to pumps, valves, etc.), and other costs.

For one house:

Average annual wastewater production = 0.48 m^3^/d × 365 days = 175 m^3^/year;
Power of drainage pump: 2.2 kW;
Pump capacity: 1.6 dm^3^/s = 5.7 m^3^/h
Annual pumping time: 175 m^3^/year / 5.7 m^3^/year = 31 h/year 31 h × 2.2 kW = 68 kWh

1 kWh = 0.7 EUR → annual cost of pumping 0 × 68 = 47.6 EUR

Operational costs of the whole system (number of damages, cost of repairs is lower than for gravity system). The total operational costs of a pressurized sewage system are equal to or lower than the gravity system. 

### 2.3. Input and Output Parameters of the Pressure Sewer System Model

A four-stage process was used to determine the quality of sewage disposal materials and systems. First, a technical analysis of pressure sewer networks in the Modlniczka village (study area in Poland) was conducted. Data from both subsystems were used to calibrate the sewage system model. In the second stage, the operation of the pressure sewer system was computed and a series of simulation studies were carried out. At the third stage, a model of the sewerage section was built in the hydrotechnical laboratory and tests were performed on the operation of the pump under leveling conditions and the cooperation of the pump with the discharge pipeline. In the final, fourth stage, sewage volume and sewage pump switching frequency were measured in a system of working pumping stations. The basic input parameters of the model were as follows:

(a) lengths of individual sections of the main pipeline,
(b) lengths of lateral sections,
(c) ordinates of nodes,
(d) ground ordinates of the pumping stations,
(e) levels of activation and deactivation of the pumping station,
(f) diameters of main pipelines,
(g) diameters of side pipelines,
(h) the diameter of the pumping station.

According to the above data, pipeline losses were calculated and pumps were designated. The parameters obtained at the output were as follows:

(a) the operating point of the pump (intersection of pump characteristics and pipeline characteristics),
(b) velocity in the various branches of the pipeline,
(c) operation time of the pumps,
(d) the number of pumps operating at the same time.

Assumptions for all simulations were a media density of 1.1 kg/dm^3^, a media temperature of 20 °C, an effluent suspended solids content of 500 mg/dm^3^, and an effluent after passing through a knife pump. Such method allows for simulations to be conducted under repeatable conditions.

### 2.4. Description of the Research Model

In the study, we used a computer program that evaluate modeling and the operation of pressure sewer systems. It was arrogated that for the studied system the time interval (time step) between successive calculation steps would be 10 s. For each pumping station (well), the daily amount of wastewater flowing into it and the type of daily distribution characteristic of each pumping station were assigned. The critical distribution was characterized by increased inflow of wastewater to the pumping station in the morning and evening hours. From the intersection of the pipeline characteristics, calculated from a given pumping station to the expansion well, and the pump characteristics, the pump discharge (Q_pump_) was obtained. As described above, the pumping well dimensions, on and off levels were fixed for the type of simulation. A distribution of water (wastewater) consumption with fairly flat characteristics can be used for the program, where the maximum hourly inflow of water (wastewater) to the pumping well is 7.9% (residential template), and it is possible to analyze a pumping station with a plant operating on a three-shift, two-shift or single-shift basis. In the case of a pressure sewer study, a critical distribution with the structure described above is included. The program anticipates that water consumption and sewage inflow to the manhole occur in the same hourly interval. The software does not analyze non-return water consumption.

**Figure 2:**
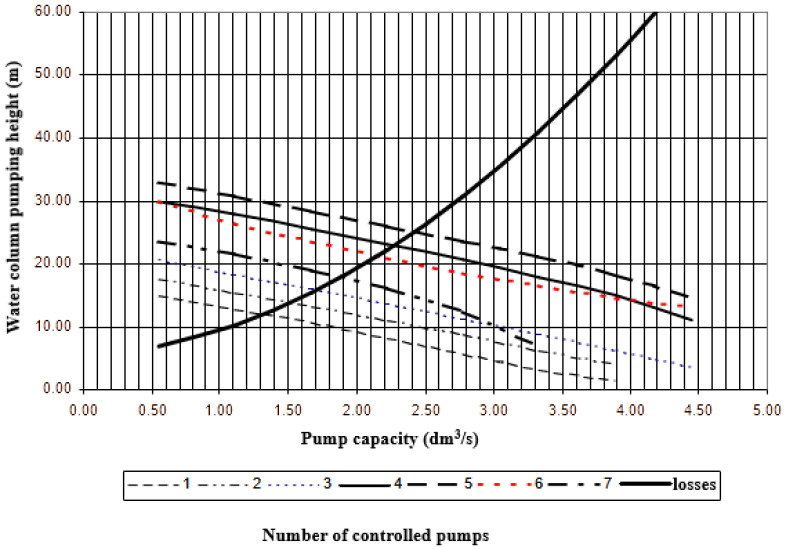
The relationship between pump capacity and water column height.

Upon entering the parameters of all pumping stations, a computer simulation of pressure sewer operation was run. The simulation started with a random level of wastewater in the manhole (the computer randomizes the value between pump on and pump off levels). With this solution, the situation is prevented, threatening to turn on an extended amount of wastewater in each pumping well uniformly reaching the level at the same or comparable time. As a result of each simulation at the pumping station, information on the day, time and length of time was obtained using relationship between pump capacity and water column height (Figure 2). In this case, a span of 30 days was adopted as the authoritative test period. The outcomes for any time interval studied are presented in graphical form on a chart, where the time and length of switching on each pumping well at hourly time intervals are highlighted.

With the observation narrowed down to a few hours, it is possible to examine the area of the system where the pumps are turned on relatively frequently for long periods. Reaching an emergency level in a given pumping well interrupts the simulation, which would be considered a system collapse. The program then allows the user to change all parameters on the well, i.e., pump capacity, well diameter, pump and pump height. In this sense, in addition to the hydraulics of the pump (operating point) and network (diameter) system itself, the program enriches the procedure for sizing a pressure sewer system with elements of sizing the pumping station itself. Normally the network is equipped with the same pump sumps, but as a result of increased sewage inflow, the sump itself needs to be replaced. In addition, the fact that in the case of centrifugal pumps the equipment reduces the capacity of the pipeline may entail a change in either the type of pump or the size of the retention, so that the pump does not reach its emergency levels.

### 2.5. Leveraging Model

During the design of the pressure sewer system, leveling occurred within the system and was verified as being a safe and secure process. This occurs when a pump pumps wastewater into a network whose elevation system causes the wastewater to be pumped upward and then transported downward until the outlet of the wastewater to the expansion sump is below the level of the pump sump. If and when the pump shutdown level is reached, due to the levitation phenomenon, the wastewater from the sump is sucked out of the domestic pumping station.

When dealing with pressurized sewers, it is important to consider whether the pump output is less than that of the equalization system or more. If it were smaller (Q_p_ < Q_lev_), more suction would be imposed on the operation of the pump. The consequence of this would be that the pump would enter into oscillation, shifting its operating point towards operation under load (from the point of view of pump selection, there would be a shift of the operating point outside the pump characteristics). A possible consequence of such a phenomenon would be damage to the pump. The hydraulic system from pump manhole 141 to expansion manhole 300 was analyzed as the network was sized, and a leveler model was made. In the first part of the study, mathematical calculations were made by numerically solving the following system of equations. For this scheme, the equation of the left branch (from the valve manhole to the leveler elbow) was of the form (1):

(1) $$\frac{P_{a}}{\gamma }=\frac{P_{k}}{\gamma }{+h}_{k}+\left(\zeta_{l1}+\lambda \frac{l_{1}}{d_{1}}+1\right)\frac{v_{1^{2}}}{2g}$$

The equation of the vertical branch (from the inflection point to the collection well) was calculated as follows (2):

(2) $$\frac{P_{k}}{\gamma }{+h}_{\mathrm{st}}-\left(\zeta_{l2}+\lambda \frac{l_{2}}{d_{2}}+1\right)\frac{v_{2^{2}}}{2g}=\frac{P_{a}}{\gamma }$$

The equation of the entire system between the pumping well and the expansion well had this mathematical formula (3):

(3) $$\frac{P_{k}}{\gamma }{+h}_{\mathrm{st}}-\left(\zeta_{l2}+\lambda \frac{l_{2}}{d_{2}}+1\right)\frac{v_{2^{2}}}{2g}=\frac{P_{a}}{\gamma }$$

where:

h_st_ = HKZ—HPTT, [m]
ζ_l1,l2_—local loss per length, [-]
λ—loss per length according to Colebrook and White, [-]
Pa—atmospheric pressure, [Pa]
Pk—pressure at the leveler knee, [Pa]
HKZ—ordinate of the sewage water table in the pumping well, [m]
HPTT—ordinate of the water table in the manhole, [m]
ΣH—sum of hydraulic losses of the leveler, [m]

Calculations have shown that the suction volume was Q = 2.41 dm^3^/s while the pumping alone, calculated in the mathematical way, was Q = 2.15 dm^3^/s.

A model of the pumping system used to determine the operation in the equalization system. The model consisted of a pump house tank, a submersible pump equipped with a cutting knife, piping made of polymethylmethacrylate (PMMA clear extruded), vent valves distributed along the route, a pressure gauge, a control valve and a measuring tank.

The pumping station’s tank was built of steel, while the front side was hardened glass, which allowed observation of the liquid level in the tank and the pump’s operation. A measurement of the water table height was introduced in the tank. The tank was calibrated for each test so that the water level was constant, i.e., the water supply to the tank was equal to the pump output. The amount of water supplied to the tank was transferred via a control valve. The tests were conducted on clean water at a temperature of about 12 °C.

A cutting knife pump of the TP40s/25 series (PolyPump Limited, Wallingford, UK) supplied with three-phase current was used for the tests. The pump was mounted in the tank on a steel rack, while the control box was mounted on the wall. A pipeline for technical reasons was connected to the pump’s discharge port, which was made in a 25-cm section from copper and then passed into PMMA. The pipeline was made of PMMA, so that the leveler operation, incomplete section flow and jet interruption could be observed. The pipeline was made of pipes with an outer diameter of 30 mm and an inner diameter of 26 mm. The individual pipeline sections were welded to each other, and the fittings were heat profiled. Aeration valves were used at the highest points of the route.

Simultaneous operation of a pressure sewer is defined as an event when more than two pumps in a pumping system have turned on and are operating continuously in any unit of time. A pressure gauge and a control valve were installed on a straight section of the pipeline downstream of the pump, allowing fluid flow to flow through the pipeline and into the well. The positions of the different openings of the valve were determined by the position of the dial mounted to the valve. The measuring tank was manufactured of steel and connected through a measuring tube made of transparent plastic. A measuring patch was installed in the measuring tube. Once the tank was calibrated, readings could be taken accurately on the measuring patch. Flow tests were conducted for two cases. In the first, the pump ran continuously until the active volume of the upper tank was completely pumped—pumping up to the height of the pump’s mechanical seals. The second was used only to create suction for the leveler, which was then turned off and the pump stopped working.

### 2.6. Field Tests and Statistical Measurements

Pumping station S4 was fabricated from PE, with an outer diameter of 900 mm. This pumping station was equipped with a three-phase pump of the TP40 type. The second pumping station, S6, was equipped with two TP40 pumps operating alternately in the same direction, each with a diameter of 1100 mm (200 mm). Readings were taken daily in the afternoon on a recorder mounted in the control cabinet. Empirically, volumes were determined using a single pump. The smoothness of pump operation was also observed. The study was conducted on a monthly basis taking meticulous daily readings. There were no power outages or failures at any of the pumping stations during the study period. The tests were conducted on domestic wastewater. The operation of a pressurized sewage system was observed, with two pumping stations operating on a common manifold. Based on the tests, it was determined that black and gray wastewater from a building inhabited by nine people flowed into pumping station S4, and wastewater from a teaching and administrative building was discharged into pumping station S6. The active volume per pumping station was determined empirically for each pumping station. The on and off levels of the pumping stations were measured from the recorder.

Interclass Correlation Coefficient (ICC) was applied to measure agreement between two or more quantitative variables (expressed numerically). ICC values above 1 indicate a higher agreement between measurements. It is possible to have a negative ICC. In this study, a one-way random effects model ICC (1,1) was used. A nonparametric measure of rank (Spearman’s correlation) was selected to show examined variables describing the quality of a pressure pump service. The calculations were analyzed using PAST version 4.11.

## 3. Results

### 3.1. System Running Time

Observations were conducted at 20, 30, 40, 50, 75 and 100 pumping stations, equipped with centrifugal pumps, operating on a common pipeline and serving a single water line. Two systems with different average inflows of wastewater to the pumping stations were studied. The values of average inflows for the two models are shown in Table 2.

In each case, observations were first made for a pumping station with an active volume of 148 dm^3^, and then the study was repeated for 296 dm^3^.

Table 2 displays the theoretical operating time of a pumping system consisting of n number of pumps The results of the operating time of the simulated system, for different active volumes (SIM1, SIM2), followed by the value of the extension of the running time of the system expressed in percentage. It can be noted that, in general, as the number of pumps in the system increases, the performance parameter for the simultaneous working of n pumps also rises (namely, the extension of time expressed in percentage). The system, composed of 100 pumping stations, with an average daily wastewater inflow of 705 dm^3^ per individual pumping station, lasted an average of 18.4–19.1% more hours. Over 30 days, the system has been running more than 50 h longer due to a decline in pump capacity resulting from simultaneous joint channel service. On a per-day basis, the average value of extended system performance was 1.4 h.

The LINK type simulation recorded an overall increase in system performance of more than 10% for pumping systems consisting of 50, 75 and 100 pumping stations (one simulation with 40 pumps). In contrast, the Type 00 simulation reported an upward rate of no more than 10% for a pressure sewer system composed of 100 domestic pumping stations.

For SIM_00 and SIM_DOUBLE simulations, the theoretical operating time was compared with the results obtained during simulation studies. The relationship is shown in Table 3. As the average capacity and number of pumps in the system increases, the system’s operating time becomes extended. There were no significant differences in the extension of system operation due to the volume settings of active pumping stations.

### 3.2. Simultaneous Co-Working

**Figure 3:**
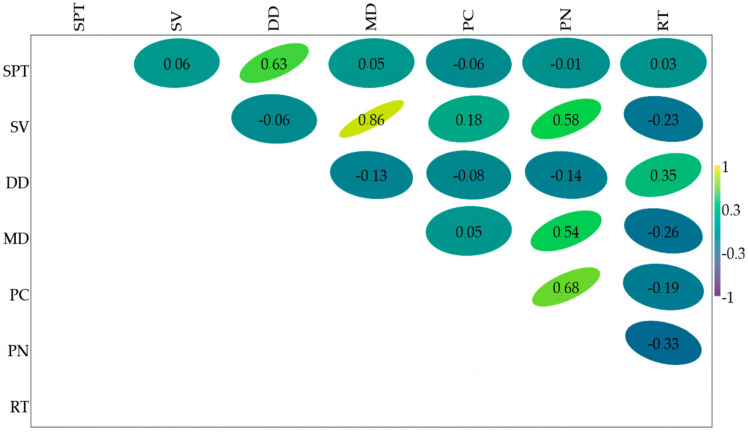
Pearson’s linear correlation for a selection of wastewater parameters of pressure system pump performance. Abbreviations of the measured variables: separation pumping time (SPT), sewage volume (SV), daily discharge (DS), monthly discharge (MD), pump capacity (PC), pump number (PN) and run-time (RT).

Pump number 1 and pump number 4 were turned on at t = 0. At pumps 1 and 4, 10 s after the first pump was added, pump number 5 was added to the mixture. Within 30 s, pump number 1 was turned off, while pumps number 4 and 5 continued to work. Pump number 2 still worked. Assuming that the pumps were turned on at the 0-s moment and off at the 30-s moment, it can be concluded that four pumps were working simultaneously, and the operating time for the described event was 30 s (approximately one second after the first pump was turned off). The observed results covered a period of 30 days. It was observed that as the number of pumps in the system increases, the number of simultaneous events grows. The frequency of simultaneous events was higher for pumping stations with a smaller active volume than for pumping stations with an active volume of 296 dm^3^. Similarly, the maximum and average times of simultaneous operation generally tended to increase as the number of observed pumps in the system increased. The examination of the parameters of simultaneous operation made it possible to note that the number of events was significantly higher for tests conducted at 148 dm^3^ active volumes. It should be pointed out that the number of simultaneous operations has consistently risen for systems with 75 or 100 pumping stations in each state. The proportion of simultaneous pumping station employees was also on average higher than the wastewater inflows to the system’s main pumping station over the same period. Pearson correlation coefficient was highest between sewage volume and monthly discharge (Figure 3).

In SIM_00 simulations for active volumes of 148 dm^3^ and 296 dm^3^ with pumping systems of 20 and 30 pumps, the share of simultaneous operation in total system operation was low, ranging from 0.5–1.1%. The results were consistent across all systems. For 40–50 days, an increasing proportion of simultaneous time was observed during system operation. In the SIM_00 simulation, it ranged from 1.7% to 2.3%, and for studies with a higher wastewater inflow (SIM_LINK), the values varied from 2.5% to 4.3%. As noted in systems with a substantial number of pumps, 75 and 100 units, simultaneous operation time accounted for a considerable percentage of the total running time of the pumping stations studied in this study. The values ranged from 3.6 to 7.0% for the SIM_LINK simulation, and for the SIM_LINK study, the values were 4.5% and 7.9% for the 75-pump system, and 12.0% and 12.1% for the 100-pump stations. The number of events for pumping wells with an active volume of 148 dm^3^ was higher than for pumping wells with a higher retention of 296 dm^3^. With the same amount of inflowing wastewater, the pumping station with the smaller active volume was emptied more often. The values obtained were a percentage of the number of events from a specific simulation that were observed in consecutive hourly time intervals. Although the number of events varied, the percentage analysis enabled a clear indication of the morning and evening peaks in the occurrence of pump switching. It is noteworthy that regardless of the type of simulation, high values for the percentage of sewage pump switch-on occurred in the morning peak, between 6 and 9 a.m., and in the evening peak, between 5 and 9 p.m.

The quantitative analysis of simultaneous operations in the SIM1_75_00, SIM2_75_00, and SYM2_75_LINK studies showed that the incidence changes as the amount of wastewater flowing into the pumping station increases, and as the active volume of the pumping station decreases, resulting in the pumps being switched on more frequently. In the SIM1_75_00 model, the simultaneous operation was observed 410 times during the 30-day period studied, while in the SIM2_75_00 simulation with a larger active volume, this event occurred 315 times during the 30-day period studied. With enhanced wastewater inflows, simultaneous operation in the SIM1_75_LINK study occurred 228 times, while in the SIM2_75_DOUBLE study it was recorded 121 times (Table 4).

Table 4 summarizes the correlation between the hourly percentage inflow of wastewater to a pumping well and the hourly distribution of the occurrence of simultaneous operations, expressed as a percentage, for all wells in the same survey area. Based on the above assumptions, it can be seen that the correlation between the hourly percentage inflow of wastewater to a pumping well and the hourly distribution of the occurrence of simultaneous operations expressed as a percentage is high. Furthermore, in the case of the SIM1_75_LINK simulation, it is very high. For simulations with a smaller active volume V = 149 dm^3^, the number of occurrences increases (in the interval from 10 to 90 s, and then sharply increases), and a trace number of occurrences was observed for the interval equal to 150 s. This observation can be made for both a system with 75 and 100 pumping wells. In the simulation with an active volume of V = 296 dm^3^, the number of events associated with joint operation of the pumps in individual time intervals was lower compared to SIM1, remaining at a similar level of abundance at intervals between 10 and 180 s, with single events lasting up to 250 s. However, for the SIM1 simulation, the shape of the curves was probably the same when studying a system of 75 and 100 pumping stations, except that as the number of pumps in the system increases, the number of events involving joint operation of deep-water pumps increases. In the SIM2 simulation, the shape of the curves was similar when examining the 75 and 100 systems.

### 3.3. Determination of the Maximum Number of Centrifugal Pumps Working on a Common Pipeline

Simulation studies were carried out for systems with a frequency range from 10 to 100 and with daily wastewater inflows averaging from 450 dm^3^ to 750 dm^3^ for one pumping well. It can be seen that the maximum number of centrifugal pumps operating on a common pipeline increases with the size of the system (number of pumps) and the amount of wastewater flowing into the system (capacity). Frequency of joint operation is much higher for smaller active volumes. For example, in a system consisting of 50 pumping wells at an average inflow of 600 dm^3^/d for a pumping station in the SIM1 simulation (active volume V = 148 dm^3^) 2 pumps jointly worked about 1000 times, while in the SIM2 simulation (active volume V = 296 dm^3^) this result occurred about 530 times.

### 3.4. Research on the Operation of a Pumping Well in a Leveller System

The total length of the pipeline was 12.73 m, the height of the leveling elbow was 1.31 above the water level in the pump chamber, and the difference in the water level between the pump chamber (well) and the outlet of the measuring chamber was 2.42, and could have been kept constant if there had been an inflow of water into the pump chamber via a control valve. The front wall of the chamber was made of glass, which made it possible to observe the water level in the chamber (well) with the pump and adjust the valve so that the outflow from the chamber was balanced by the inflow of water into the chamber, and thus the water table was at a constant level. The flow was measured using volumetric methods. The system was equipped with a vent valve, a graduated butterfly valve, and a pressure gauge installed on a straight vertical section behind the pump. For each of the 6 positions on the valve-opening indicator, system expenditures and pipeline flow velocities were calculated. The trend lines for the velocities and for the expenditures were also determined and the equations of the trend lines were given. During the study of the leveling model, the local loss coefficient for the DN25 ball valve was also determined. The relationship between the change in pipe velocity and the local loss coefficient is nonlinear (Figure A2).

### 3.5. Field Studies

Calibration of the active volume pumped during one cycle of operation was carried out for the S4 pumping station. The level was 35 cm, the offset level was 20 cm, the count was from the bottom of the pumping station, and the pumping time for this volume was calculated to be 28 *s*. The internal diameter of the pumping station was measured and a value of 826 mm was obtained. The pumping station in the observed section was cylindrical in shape, so the active volume of 80.3 dm^3^ was calculated from simple mathematical calculations of the volume of the pump at the time of the analysis. The S6 pumping station received wastewater in a larger volume, so the design stage assumed the use of submersible wastewater pumps operating alternately to ensure reliable operation of the system with a pumping well of similar volume. For pumping well S4, the average daily volume of incoming sewage was 1225.1 dm^3^, which, per capita, gives an average consumption of 136 dm^3^ per day. The S6 pumping well recorded an average daily volume of inflowing sewage equal to 5926.6 dm^3^. The minimum observed value was 642.7 dm^3^ for 1 day, while the maximum value was almost three times higher, equal to 1847.75 dm^3^. The high variability of the amount of incoming wastewater in pressure channels affected both the cooperation of pumps and the amount of wastewater exchanged in the pipelines. With such large diurnal irregularities, it would be difficult to select systems in such a way as to maximize the retention time of wastewater in the pipeline on the one hand, and avoid frequent throttling of the system on the other. For the aforementioned pumping station, the average daily volume of inflowing wastewater was 5926 dm^3^, while the daily maximum volume was read as 7840 dm^3^. Confidence intervals from −0.95% to +0.95% were determined for both pumping stations. Noteworthy is the fact that during the 24 days of observation, the pumping stations pumped, respectively, 29.403 dm^3^ (pumping well S4) and 14.2240 dm^3^ (pumping well S6).

## 4. Discussion

### 4.1. Sewage-Treatment Management

An important aspect in the selection of a sewer system is the economic analysis of the investment. The ratio of capital intensity of pressure sewer construction for a 150-m pipeline in dry soils is twice that of gravity sewers. For severely irrigated soils, the differences are even more pronounced [30,31,32,33,34]. Studies on the dynamics of sewage inflow to gravity sewer systems indicated a large infiltration of rainwater into the sewer system. In pressurized sewers, infiltration water inflow is negligible [35]. The issue of accurately determining the amount of wastewater discharged into the system is extremely important, especially for pressure and pressure sewerage systems.

In pressure sewer systems, an extremely important part of the design is the calculation of the number of pumps that can operate at the same time on a common pipeline [36]. Submersible sewage pumps with a free passage of 80 mm are adapted to pump a capacity equal to Q = 25 dm^3^/s to an overall height equal to 20 m, centrifugal pumps equipped with a cutting knife can pump smaller capacities, where Q = 3 dm^3^/s, but to an overall height reaching 26 m, and analyzing the left side of the pump characteristics, the height reaches up to 40 m. The operating point of a positive displacement pump having a capacity of Q = 0.66 dm^3^/s and a total head of H = 55 m is recommend.

Some designers have assumed that a maximum of 1/3 of all pumping stations operating in the system can be turned on, but this is not the case. Designing for such parameters in the case of centrifugal pumps could lead to system inefficiency. Number of simultaneously operating pumps in a pressurized sewer system can be determined by computer mathematical models, on the basis of probability calculus, or by using quantities obtained from operating systems (Table 3). Using probability calculations or simulating the flow of sewage into each domestic pumping station can be used to estimate the number of working pumps within the sewerage system (Table 3). Simulating the pumping operation of individual stations is recommended, especially when each station is switched independently (no automatic transmission of data on the status of the pumping stations).

When comparing the centrifugal pump characteristics to those of the positive displacement pump, it becomes evident that the centrifugal pump has much fewer steep characteristics (Figure A2). A positive displacement pump system will decrease capacity slightly when several devices working in it are switched on, while a centrifugal pump system will decrease capacity dramatically. Despite the short period of observation between sewage pumping stations S4 and S6, a large daily variation in sewage inflow was observed. These variations are so large that it would be technically and logically difficult to develop a simulation model that takes into account the diurnal variation in the volume of inflowing wastewater (Table A1). There is some debate as to whether or not intuition and experience should play a larger role in the selection of pressure pipes used in pressure sewerage. Based on our research, computer programs and simulations should be developed. The volumes of actual inflowing wastewater are specific to a given facility or system and may not fully coincide with design assumptions (Table A2). The inflows to the pressure-sensitive sewer system can only be evaluated and verified with the actual parameters of the long-term investment [37]. Each pressurized sewer system will be characterized by slightly different dynamics of inflow to the pumping wells [38].

More elaborate systems may experience significant consequences from the interaction of dozens of pumping stations on a common pipeline. The use of pressure systems is particularly attractive when the terrain layout is unfavorable, development is scattered, mining subsidence occurs, and long-distance pumps are required. This makes gravity sewers either economically or technically challenging [39]. Moreover, such systems should be considered if groundwater levels are too high, sewerage systems require special tightness as follow:

- great freedom in routing sewer routes;
- possibility of cooperation with other systems, such as gravity sewers or pressure systems;
- elimination of infiltration and exfiltration phenomena through the need to use sealed pressure pipes;
- potentially lower construction costs of pressure sewer systems in comparison with sanitary sewer systems due to shallower excavations [1.5 m] and smaller pipe diameters [from DN 63].

### 4.2. Reducing the Leveling Effect in Rural Sewage Systems

As demonstrated by field studies conducted on low pressure sewer systems and studies by other authors, rural sewer systems are characterized by high variability in wastewater inflow both daily and hourly (Tab 3). In the case of studies of large systems consisting of dozens of pumping stations, in-line sewage inflow measurements would have to be made to obtain meaningful results. The results were not immediately available. It is also worth noting that in the case of extensive systems, a gigantic amount of data would have to be acquired, and the values obtained would also be heavily influenced by random events that are difficult to isolate and interpret. Such research would be extremely expensive, and the ability to generalize the results of the research and draw conclusions about the broader issue would be significantly limited [40]. The following simplifications were used in the research on the computer model:

- the daily sewage inflow to each pumping well was randomly drawn from a specific interval, but during the simulation studies after the drawing it was constant for the entire study period (30 days);
- the hourly distribution of wastewater inflow for each day of the entire study period considered;
- the density was assumed to be 1.1 g/cm^3^, viscosity 1 mm^2^/s, medium temperature 20 °C, suspended solid content 500 mg/dm^3^, according to Fyodorov’s tests.

It was assumed that the pumps operate ideally according to the characteristics included in Figure A4 (in practice, depending on the type of pump, deviations in the operation of equipment are allowed). The use of the computer model made it possible to create comparable conditions for systems consisting of 20, 30, 40, 50, 75 and 100 pumping stations. Such a solution also made it possible to observe the operation of the pressure sewer system with a random but controlled increase in sewage inflow to selected pumping stations [41].

The leveler model built in the laboratory, which is a representation of a fragment of the system in studied municipality, allowed analysis of the operation of sewage pumps under levelling conditions. For the SIM_75_00 and SIM_75_LINK simulations studied, with active volumes V = 148 dm^3^ and V = 296 dm^3^, a high correlation was found between the time interval of wastewater inflow to the pumping stations and the percentage and quantity occurrence of simultaneous operation. The correlation coefficient ranged from 0.54 to 0.72 (Table 3). The lowest Pearson correlation coefficient appeared between the number of pumps and run-time (Figure 3). With the assistance of model research, the performance of large pumping systems was studied and the interaction of pumps was determined. From the number of pumps, the amount of wastewater flowing into the pumping station and the active volume of wastewater flowing into the pumping station, the pressure extension time of the sewer system was calculated.

It was found that active volume had little effect on total system operation length, but it had a significant impact on the number of pumps operating at the same time (Figure A3). Moreover, it was proven that the maximum number of pumps working at the same time is significantly influenced not only by the active volume of one pump, but also by the amount of sewage flowing into each pumping well [42].

Plenty of emphasis has been placed on the impact of pressure-sensitive sewer operations under elevated conditions. As a result of a nonlinear relationship between the local loss coefficient of a ball valve in the leveling system and the flow rate at 5.5 m/s for the opening position 6 of the valve, the coefficient is 2, while for the minimal opening position 1, it is 72 at 0.8 m/s. A pump output greater than a leveler output is required for the leveler-pump system to work properly. The results indicated that it is important to measure the velocity and discharge levels at different degrees of openings in the ball valve installed at the pumping manhole. We believe that it is necessary to determine the local loss coefficient for this valve and the resistance coefficient of the pump under study in order to determine the local loss coefficient. Studies of the performance of the pump sump in the leveling system have shown that if the pump capacity is greater than the standard pump sump the pumping system will work properly. In contrast, a pump with a lower capacity than the leveler sucked wastewater from the pumping well.

### 4.3. Future Recommendation

The article describes how the volume of incoming wastewater and terrain affects the operation of pressurized sewers. These volumes can be categorized as parameters independent of the designer. The designers can influence the system by using different volumes of active pumping wells and pumps with different operating characteristics, as well as by dividing a large pumping station into smaller, independent subsystems from which a larger pumping system can be generated. The listed parameters have been considered critical parameters, i.e., those that have a significant impact on the proper functioning of the sewerage system, and which, if ignored, can threaten to collapse the system. Work is still needed to study the pressure sewers for their use in the sewerage of rural settlement units. An important postulate is the implementation of updated design guidelines that take into account the current state of knowledge and technology [43,44].

According to the frequency of engagements performed on pumping wells, pumps with a smaller active volume (SIM1) have a significantly higher number of engagements than pumps with a larger active volume (SIM2). The determined resistance coefficient of the tested sewage shut-off pump was equal to 0.93. Field tests of two sewage pumping stations operating on a common manifold showed a high diurnal variability in the inflowing of sewage. At pumping station S4, the minimum daily amount of wastewater was 642 dm^3^. The maximum was 1847 dm^3^. In the case of pumping station S6, the daily amount of inflowing sewage is much higher, and this volume requires the use of 100% pumping reserves.

Hitherto, the pressure system has been studied from the point of view of a single pumping well. It seems innovative to examine the performance of a pressure sewer system as a unit [45], involving economic factors [46,47]. In order to understand this issue, the concept of simultaneous system operation is presented as a set of basic statistical methods for identifying the underlying data. The results indicated that the load state of the entire system is relevant for assessing technical concerns. The length of the pump run has a less considerable effect. Expansion of active volume leads to increased simultaneous operation times, and the duration of events varies with the amount of wastewater flowing into the pumping wells. Studies have shown that there is a significant correlation between hourly wastewater inflow and the occurrence of simultaneous operations (Table 3). We can suppose that the retention of pumping wells is a factor that limits the impact of wastewater inflow on simultaneous operation events (there is a time delay in the switching of pumping wells). An essential element, not discussed in any literature, is the possibility of determining how to create a particular aspect of the lifting system. Controlling the permeability of the descending branch of the equalization system is vital to improving hydraulic resistance to the current of the equalization system. When the permeability of the descending portion is higher, all effluent corresponding to that permeability will be sucked into the system. This increases the suction coefficient and enables the diameter to be reduced in size.

## 5. Conclusions

As a result of these experiments, it was concluded that the simultaneous operation of more than 75 pumping stations has a significant impact on the overall pumping capacity and efficiency of large systems. An extensive system investigation and complete simplification of the modeling phase were a prerequisite for full-scale implementation in the area under study. A 100-pump system can run simultaneously for 12% of the total operating time. The model has a limitation in terms of the number of pumping stations. It was found that the number of simultaneous operations was dependent on the number and size of active pumping stations. A village’s maximum capacity should be divided into subsystems based on its size. Subsystems with fewer than 100 pumping stations were not considered defective. The leveler-pump system requires a pump with a capacity greater than that of the leveler. For the full opening of the ball valve for the model system, a standard capacity of 0.7474 dm^3^/s was obtained. Increasing the active volume at the pumping station does not significantly affect the extended operation of the system. The operation of a pressurized sewerage system depends on a number of parameters, including the amount of wastewater supplied to the pumping wells. This includes the monthly inflow volume at a specific hour, the active retention volume, and the number of pumps functioning simultaneously. Additionally, the rate at which a device runs simultaneously affects its periodic self-cleaning. Predictability and difficulty are the biggest challenges in pressure sewer planning. Future studies should determine the volume of wastewater flowing into the pumping station on a daily basis.

## Figures and Tables

**Table 1:** The parameters of model pumping stations.

		Simulation 1	Simulation 2
Inner diameter	m	0.78	0.78
H inclusion	m	1.29	0.98
Depth	m	1.8	1.8
H exclusion	m	1.6	1.6
H emergence	m	1.24	0.90
Volume of one pumping	dm^3^	148.05	296.11

**Table 2:** The values of average wastewater inflows to a single pumping station depending on the type of study and the number of pumps in the system.

Number of Pumps in a System	20	30	40	50	75	100
	dm^3^					
SIM 00	499	477	463	462	458	468
SIM LINK	752	719	695	680	692	705

**Table 3:** The theoretical and simulation of pumping systems for 148 dm^3^ and 296 dm^3^ active volumes.

Simulation: DOUBLE					
Number of Pumps in the System	Theoretical Time	SIM1	SIM2	SIM1	SIM2
				Time extension	
	s			%	
20	179,711	191,612	190,151	6.2	5.5
30	243,843	267,183	264,139	8.7	7.7
40	306,567	340,846	339,513	10.1	9.7
50	374,199	425,117	422,075	12	11.3
75	561,521	673,872	683,197	16.7	17.8
100	800,357	981,394	989,314	18.4	19.1
Simulation: 00					
20	113,840	124,020	122,580	8.2	7.1
30	163,232	169,380	173,520	3.6	5.9
40	211,255	220,200	221,520	4.1	4.6
50	263,498	278,640	282,060	5.4	6.6
75	391,825	424,560	425,640	7.7	7.9
100	533,840	625,980	623,940	14.7	14.4

**Table 4:** The correlation between the inflow of wastewater to the pumping well and the hourly distribution of the occurrence of simultaneous operation expressed in percentage.

							Time Interval
%	Number of Incidents	%	Number of Incidents	%	Number of Incidents	%	s
SIM2_75_00		SIM1_75_00		SIM2_75_LINK		SIM1_75_LINK	
1.7	2	1.3	3	0.6	2	1.5	5–6
7.4	9	6.6	15	7.3	23	5.4	6–7
10.7	13	5.7	13	6.7	21	11.7	7–8
5.8	7	3.5	8	4.1	13	3.7	8–9
2.5	3	2.6	6	1.3	4	2	9–10
2.5	3	1.8	4	1.3	4	0.5	10–11
2.5	3	2.6	6	1.3	4	0.5	11–12
0.8	1	2.2	5	3.5	11	1.5	12–13
2.5	3	1.3	3	2.9	9	1	13–14
2.5	3	0.4	1	2.2	7	2.2	14–15
1.7	2	3.1	7	2.5	8	1.2	15–16
0.8	1	3.5	8	5.4	17	4.9	16–17
5.8	7	4.4	10	3.8	12	6.3	17–18
19.8	24	17.1	39	16.2	51	16.8	18–19
21.5	26	21.1	48	17.5	55	13.4	19–20
7.4	9	6.1	14	11.1	35	12.4	20–21
2.5	3	10.1	23	7.6	24	9.3	21–22
1.7	2	6.6	15	4.8	15	5.9	22–23
100	121	100	228	100	315	100	Total
0.54		0.63		0.69		0.72	Correlation coefficient

ICC (2, 1) for individual 0.021; confidence interval: (0.0016; 0.051); ICC (2, k) for mean 0.13; confidence interval: (0.012; 0.276).

## Data Availability

Data are contained within the article.

## Appendix A

**Table A1:** Simultaneous operation parameter listings for a pumping station with V = 148 dm^3^.

Simulation Type	SIM1_10	SIM1_20	SIM1_30	SIM1_40	SIM1_50	SIM1_750	SIM1_100
Sum [s]		560	1860	3660	4910	15,160	43,546
Maximum [s]	No data	100	140	130	180	170	270
Average [s]		56	60	64	65	66	79
Number of events		10	31	57	76	228	544
Dail flow [dm^3^]		499	477	463	462	458	468
Simulation type	**SIM1_11**	**SIM1_21**	**SIM1_31**	**SIM1_41**	**SIM1_51**	**SIM1_751**	**SIM1_101**
Sum [s]		510	1770	2080	6190	18,230	40,203
Maximum [s]	No data	130	100	190	160	250	270
Average [s]		73	63	58	64	71	76
Number of events		7	28	36	96	257	527
Dail flow [dm^3^]		525	490	473	471	465	470
Simulation type	**SIM1_12**	**SIM1_22**	**SIM1_32**	**SIM1_42**	**SIM1_52**	**SIM1_752**	**SIM1_102**
Sum [s]	150	980	1530	3840	7540	15,650	50,403
Maximum [s]	90	100	150	170	380	200	280
Average [s]	75	65	61	59	67	67	82
Number of events	2	15	25	65	112	233	617
Dail flow [dm^3^]		545	508	479	476	473	476
Simulation type	**SIM1_13**	**SIM1_23**	**SIM1_33**	**SIM1_43**	**SIM1_53**	**SIM1_753**	**SIM1_103**
Sum [s]	180	890	1760	4300	5850	17,300	42,046
Maximum [s]	70	110	110	210	240	170	340
Average [s]	45	64	61	70	64	66	84
Number of events	4	14	29	61	92	261	501
Dail flow [dm^3^]		574	527	494	488	481	482
Simulation type	**SIM1_14**	**SIM1_24**	**SIM1_34**	**SIM1_44**	**SIM1_54**	**SIM1_754**	**SIM1_104**
Sum [s]	210	860	2440	4470	6800	22,010	44,489
Maximum [s]	70	120	150	190	260	230	210
Average [s]	53	66	61	67	67	73	76
Number of events	4	13	40	67	101	303	583
Dail flow [dm^3^]		601	547	507	500	488	486
Simulation type	**SIM1_15**	**SIM1_25**	**SIM1_35**	**SIM1_45**	**SIM1_55**	**SIM1_755**	**SIM1_105**
Sum [s]	400	1600	2570	3570	7260	20,960	45,774
Maximum [s]	110	160	180	150	150	300	260
Average [s]	57	73	73	57	60	70	85
Number of events	7	22	35	63	122	300	536
Dail flow [dm^3^]		621	559	521	509	495	492
Simulation type	**SIM1_16**	**SIM1_26**	**SIM1_36**	**SIM1_46**	**SIM1_56**	**SIM1_756**	**SIM1_106**
Sum [s]		1380	2830	5000	8850	22,420	43,589
Maximum [s]	No data	130	100	170	270	250	130
Average [s]		63	63	60	65	73	81
Number of events		23	45	84	136	306	540
Dail flow [dm^3^]		644	575	534	519	502	498
Simulation type	**SIM1_19**	**SIM1_29**	**SIM1_39**	**SIM1_49**	**SIM1_59**	**SIM1_759**	**SIM1_109**
Sum [s]		1900	3520	5570	10,130	23,640	48,089
Maximum [s]	No data	110	220	170	150	290	210
Average [s]		68	72	66	65	72	81
Number of events		28	49	85	157	329	596
Dail flow [dm^3^]		725	621	565	544	522	514
Simulation type	**SIM1_10p**	**SIM1_20p**	**SIM1_30p**	**SIM1_40p**	**SIM1_50p**	**SIM1_75p**	**SIM1_100p**
Sum [s]		2380	4340	8440	15,860	31,050	118,594
Maximum [s]	No data	190	130	150	220	280	330
Average [s]		79	64	64	69	75	94
Number of events		30	68	131	229	412	1256
Dail flow [dm^3^]		752	719	695	680	692	705

**Table A2:** Simultaneous operation parameter listings for a pumping station with V = 296 dm^3^.

Simulation Type	SIM2_10	SIM2_20	SIM2_30	SIM2_40	SIM2_50	SIM2_750	SIM2_100
Sum [s]		650	1120	5100	6180	17,480	39,817
Maximum [s]	No data	170	180	320	240	410	430
Average [s]		130	124	138	131	144	157
Number of events		5	9	37	47	121	253
Dail flow [dm^3^]		499	477	463	462	458	468
Simulation type	**SIM2_11**	**SIM2_21**	**SIM2_31**	**SIM2_41**	**SIM2_51**	**SIM2_751**	**SIM2_101**
Sum [s]	160	450	1360	2740	7000	21,500	34,845
Maximum [s]	160	200	190	260	250	450	530
Average [s]	160	113	113	144	127	148	159
Number of events	1	4	11	18	54	144	214
Dail flow [dm^3^]		525	490	473	471	465	470
Simulation type	**SIM2_12**	**SIM2_22**	**SIM2_32**	**SIM2_42**	**SIM2_52**	**SIM2_752**	**SIM2_102**
Sum [s]	520	1210	1420	3720	6100	20,780	34,331
Maximum [s]	180	200	190	260	230	530	530
Average [s]	173	151	142	133	122	159	141
Number of events	3	7	9	27	49	130	240
Dail flow [dm^3^]		574	527	494	488	481	482
Simulation type	**SIM2_13**	**SIM2_23**	**SIM2_33**	**SIM2_43**	**SIM2_53**	**SIM2_753**	**SIM2_103**
Sum [s]	60	1100	2390	3460	7840	21,730	53,275
Maximum [s]	60	180	200	370	370	490	430
Average [s]	60	138	114	138	140	150	173
Number of events	1	7	20	24	55	144	304
Dail flow [dm^3^]		574	527	494	488	481	482
Simulation type	**SIM2_14**	**SIM2_24**	**SIM2_34**	**SIM2_44**	**SIM2_54**	**SIM2_754**	**SIM2_104**
Sum [s]	150	1060	2500	3420	7940	23,310	41,746
Maximum [s]	150	200	260	180	380	420	450
Average [s]	150	133	132	127	137	147	162
Number of events	1	7	18	26	57	158	253
Dail flow [dm^3^]		601	547	507	500	488	486
Simulation type	**SIM2_15**	**SIM2_25**	**SIM2_35**	**SIM2_45**	**SIM2_55**	**SIM2_755**	**SIM2_105**
Sum [s]		1200	3270	4090	8380	25,810	45,217
Maximum [s]	No data	180	350	290	300	560	610
Average [s]		109	142	128	131	155	170
Number of events		10	22	31	63	166	261
Dail flow [dm^3^]		621	559	521	509	495	492
Simulation type	**SIM2_16**	**SIM2_26**	**SIM2_36**	**SIM2_46**	**SIM2_56**	**SIM2_756**	**SIM2_106**
Sum [s]		500	3010	3710	11,030	24,050	57,732
Maximum [s]	No data	160	270	190	250	400	630
Average [s]		160	151	112	140	146	185
Number of events		4	19	32	78	164	309
Dail flow [dm^3^]		644	575	534	519	502	498
Simulation type	**SIM2_19**	**SIM2_29**	**SIM2_39**	**SIM2_49**	**SIM2_59**	**SIM2_759**	**SIM2_109**
Sum [s]		1820	4140	5940	10,940	26,400	49,503
Maximum [s]	No data	230	230	270	310	380	530
Average [s]		121	134	145	146	150	178
Number of events		15	31	41	75	176	279
Dail flow [dm^3^]		725	621	565	544	522	514
Simulation type	**SIM2_10p**	**SIM2_20p**	**SIM2_30p**	**SIM2_40p**	**SIM2_50p**	**SIM2_75p**	**SIM2_100p**
Sum [s]	870	1870	6050	11,320	18,550	53,300	119,108
Maximum [s]	150	290	250	290	480	600	770
Average [s]	109	141	138	138	153	165	185
Number of events	7	13	43	81	120	324	639
Dail flow [dm^3^]		752	719	695	680	692	705
